# Increasing efficiency of preclinical research by group sequential designs

**DOI:** 10.1371/journal.pbio.2001307

**Published:** 2017-03-10

**Authors:** Konrad Neumann, Ulrike Grittner, Sophie K. Piper, Andre Rex, Oscar Florez-Vargas, George Karystianis, Alice Schneider, Ian Wellwood, Bob Siegerink, John P. A. Ioannidis, Jonathan Kimmelman, Ulrich Dirnagl

**Affiliations:** 1 Department of Biostatistics and Clinical Epidemiology, Charité Universitätsmedizin Berlin, Berlin, Germany; 2 Center for Stroke Research, Charité Universitätsmedizin Berlin, Berlin, Germany; 3 NeuroCure Clinical Research Center, Charité - Universitätsmedizin Berlin, Berlin, Germany; 4 Department of Experimental Neurology, Charité Universitätsmedizin Berlin, Berlin, Germany; 5 Bio-health Informatics Group, School of Computer Science, The University of Manchester, Manchester, United Kingdom; 6 Centre for Health Informatics, Macquarie University, Sydney, Australia; 7 Department of Public Health and Primary Care, Cambridge Institute of Public Health, University of Cambridge School of Clinical Medicine, Cambridge, United Kingdom; 8 Center for Transforming Biomedical Research, Berlin Institute of Health, Berlin, Germany; 9 Meta-Research Innovation Center at Stanford (METRICS), and Departments of Medicine, of Health Research and Policy, and of Statistics, Stanford University, Stanford, California, United States of America; 10 STREAM Research Group, Biomedical Ethics Unit, McGill University, Montreal, Canada; 11 German Center for Neurodegenerative Diseases (DZNE), Berlin Site, Berlin, Germany; 12 German Center for Cardiovascular Research (DZHK), Berlin site, Berlin, Germany

## Abstract

Despite the potential benefits of sequential designs, studies evaluating treatments or experimental manipulations in preclinical experimental biomedicine almost exclusively use classical block designs. Our aim with this article is to bring the existing methodology of group sequential designs to the attention of researchers in the preclinical field and to clearly illustrate its potential utility. Group sequential designs can offer higher efficiency than traditional methods and are increasingly used in clinical trials. Using simulation of data, we demonstrate that group sequential designs have the potential to improve the efficiency of experimental studies, even when sample sizes are very small, as is currently prevalent in preclinical experimental biomedicine. When simulating data with a large effect size of d = 1 and a sample size of *n* = 18 per group, sequential frequentist analysis consumes in the long run only around 80% of the planned number of experimental units. In larger trials (*n* = 36 per group), additional stopping rules for futility lead to the saving of resources of up to 30% compared to block designs. We argue that these savings should be invested to increase sample sizes and hence power, since the currently underpowered experiments in preclinical biomedicine are a major threat to the value and predictiveness in this research domain.

## Background

Group sizes in preclinical research are seldom informed by statistical power considerations but rather are chosen on practicability [1, 2]. Typical sample sizes are small, around *n* = 8 per group (http://www.dcn.ed.ac.uk/camarades/), and are only sufficient to detect relatively large sizes of effects. Consequently, true positives are often missed (false negatives), and many statistically significant findings are due to chance (false positives). Such results lack reproducibility, and the effect sizes are often substantially overestimated (“Winner’s curse”) [2–5]. Therefore, various research bodies (e.g., National Institutes of Health, United Kingdom Academy of Medical Sciences) have called for increased sample sizes [5, 6], as well as other design improvements in preclinical research. Yet, such calls also potentially antagonize the goal of minimizing burdens on animals. Here, we propose the use of sequential study designs to reduce the number of experimental animals required, as well as to increase the efficiency of current preclinical biomedical research. Moreover, our aim with this article is to bring the existing methodology of group sequential designs to the attention of researchers in the preclinical field and to clearly illustrate its potential utility.

## Sequential study designs

Conventional study designs in experimental preclinical biomedicine use nonsequential approaches, in which group sizes are predetermined and fixed, and the decision to either accept the (alternative) hypothesis or fail to reject the null hypothesis is made after spending all experimental units in each group. In contrast, a group sequential design is a type of adaptive design that allows for early stopping of an experiment because of efficacy or futility, based on interim analyses before all experimental units are spent [7–9], thereby offering an increase in efficiency.

However, interim analyses come at a statistical cost, and special analysis methods and careful preplanning are required. Traditional frequentist statistics can be used to split the overall probability of type I error (α–error) to account for multiple testing [10, 11], but Bayesian methods are particularly suited, as they can incorporate information from earlier stages of the study. Moreover, Bayesian analysis enables the researcher to use prestudy information as a basis for the prior information about the measure of interest [8, 9]. As the prior is potentially subjective and the gained posteriors highly dependent not only on the data but also on the chosen prior, the practice of informed priors is hotly contested. Noninformative priors are an option to circumvent this concern [12, 13].

Group sequential designs are increasingly used in clinical research [8, 14]. So far, however, they are virtually nonexistent in preclinical experiments. We performed text-mining of the complete PubMed Central Open Access subset (time frame: 2010–2014) and found only one article explicitly describing an original study evaluating a treatment in rats or mice using a sequential design [15] (S1 Text).

To explore the potential for group sequential designs to increase the efficiency of preclinical studies, we simulated data for two-group comparisons of different effect sizes and compared “costs,” measured by the number of animals required for different group sequential designs, compared to a traditional nonsequential design (S1 Text).

## Increase in efficiency

**Fig 1:**
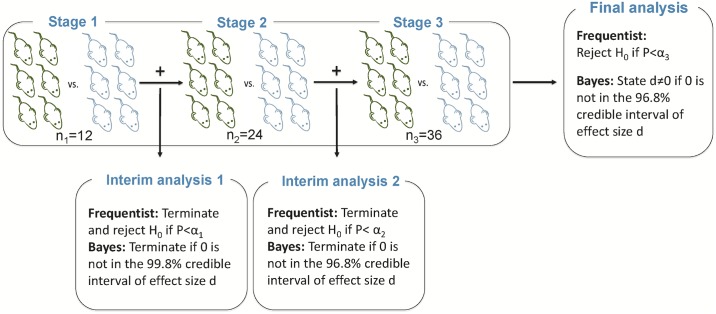
Study design and sequential analysis approach allowing two interim analyses. Stage 1: 33% of samples acquired, stage 2: 66% of samples acquired, and stage 3: 100% of samples acquired. H_0_: null hypothesis, P: *p*-value, Credible interval: specific Bayesian interval of certainty about an estimate, d: effect size Cohen’s d, α_i_: significance levels for each stage derived from [11] α_1_ = 0.0006, α_2_ = 0.0151, α _3_ = 0.0471. Additionally, we used a Bayes factor approach (Table 1) and Pocock boundaries for the frequentist approach (S1 Table). All sequential approaches used were calibrated by using simulations to get a type I error of about 5%.

We simulated a mouse experiment in which 36 animals are allocated to two groups. Currently, in most domains of preclinical medicine, group sizes of ten or less are prevalent, leading to grossly underpowered studies [4]. A group size of 18 animals per group allows the detection of a standardized effect size of d = 1, given traditional constraints of alpha = 0.05 and beta = 0.20. A block design typically used in this type of study needs to include all animals before data analysis. In a group sequential design, an interim analysis is conducted, and a predefined set of rules determine whether the experiment should be continued or not (Fig 1).

Here, we demonstrate only some of many possible analysis approaches (frequentist sequential with O’Brien–Fleming boundaries [11], with Pocock boundaries [16] [S1 Table], Bayes Factor, and Bayes credible intervals, Table 1). See Box 1 for other approaches and references.

**Table 1:** Early stopping for significance or futility using nonsequential group sequential designs (examples with *n* = 36 or *n* = 72).

	Small study (*n* = 36) stop for significance, three stages					Larger study (*n* = 72) stop for significance or futility, two stages				
	Sample size (per group)	Freq. nonseq.	Freq. seq.	Bayes Factor	Bayes (CRI) with noninf. Prior	Sample size (per group)	Freq. nonseq.	Freq. seq.	Bayes Factor	Bayes (CRI) with noninf. Prior
**d = 0**										
**Stage 1** [%] sign./futility **Stage 1 and 2** [%] sign. **Stage 1 and 2 (and 3) = type 1 error** [%] sign.	12 (6 versus 6) 24 (12 versus 12) 36 (18 versus 18)	- - **5.0**	0.1 1.4 **4.9**	2.3 4.0 **5.0**	0.4 3.2 **5.0**	36 (18 versus 18) - 72 (36 versus 36)	- **5.0**	0.8/50.7 **5.3**	3.5/70.7 **5.0**	1.1/50.1 **5.1**
**Cost** [mean number of animals] **d**_**est**_		36 0.78	36 0.84	36 1.38	36 1.00		72 0.54	53 0.55	45 0.77	54 0.57
**d = 0.5**										
**Stage 1** [%] sign./futility **Stage 1 and 2** [%] sign. **Stage 1 and 2 (and 3) = Power** [%] sign.	12 (6 versus 6) 24 (12 versus 12) 36 (18 versus 18)	- - **30.8**	0.4 10.3 **31.2**	6.4 15.3 **24.3**	1.0 18.4 **30.3**	36 (18 versus 18) - 72 (36 versus 36)	**55.3**	9.7/18.8 **53.8**	25.6/32.5 **46.0**	11.2/18.5 **54.3**
**Cost** [mean number of animals] **d**_**est**_		36 0.86	35 0.93	34 1.13	34 1.00		72 0.65	62 0.67	51 0.78	61 0.68
**d = 1.0**										
**Stage 1** [%] sign./futility **Stage 1 and 2** [%] sign. **Stage 1 and 2 (and 3) = Power** [%] sign.	12 (6 versus 6) 24 (12 versus 12) 36 (18 versus 18)	- - **83.0**	1.6 43.6 **82.2**	22.8 53.3 **74.7**	4.5 58.4 **80.5**	36 (18 versus 18) - 72 (36 versus 36)	**98.7**	54.4/0.9 **98.1**	78.3/2.8 **96.1**	57.7/0.8 **98.2**
**Cost** [mean number of animals] **d**_**est**_		36 1.09	31 1.16	27 1.27	28 1.14		72 1.01	52 1.07	43 1.05	51 1.06

**Simulations based on a total number of 18 or 36 samples per group. Power or type I error for three different standardized effect sizes Cohen’s d = 0, or 0.5, or 1.0, respectively.** Numbers give **cumulative** percentages of statistically significant trials in percent [%] out of 10,000 simulation runs, as well as “Costs” defined as the long term mean of experimental units, and median estimated effect sizes in significant trials (d_est_). **Small study with *n* = 18 per group:** Stage 1: *n* = 12 (6 versus 6), stage 1 and 2: *n* = 24 (12 versus 12), stage 1 and 2 and 3: *n* = 36 (18 versus 18) experimental units. Stopping rules that allowed early stopping: Freq. nonseq.: α = 0.05; Freq. seq.: significance levels for interim analyses: α_1_ = 0.0006, α_2_ = 0.0151, α_3_ = 0.0471 according to [11]; Bayes Factor: 3 for each stage; Bayes noninf. prior: CRI for effect size: stage 1: 99.8% CRI, stage 2 and 3: 96.8% CRI.

**Larger study with *n* = 36 per group:** Stage 1: *n* = 36 (18 versus 18), stage 1 and 2: *n* = 72 (36 versus 36) experimental units. Stopping rules that allowed early stopping for futility or significance: Freq. nonseq.: α = 0.05; Freq seq. [11]: α_futility_ = 0.5, α_1_ = 0.0065, α_2_ = 0.0525; Bayes Factor: 2 and for futility: 0.5; CRI for effect size d using a Bayesian approach with noninf. prior: stage 1 99% CRI, for futility: zero is included in 50% CRI for effect size d, stage 2 95% CRI.

All sequential approaches used were calibrated to get a type I error of about 5%.

**Abbreviations:** CRI, credible interval; Freq. nonseq., Frequentist nonsequential; Freq. seq., Frequentist sequential; Noninf., Noninformative.

The O’Brien–Fleming boundaries in the frequentist sequential approach keep the alpha level for the final analysis (stage 3) approximately as high as for the classical block design. Additionally, the same scenarios using Pocock boundaries can be found in S1 Table. It should be noted that the frequentist approaches refer to null hypothesis significance testing, whereas the Bayes Factor approach is basically a model comparison, and the other Bayesian approach uses credible intervals for estimates. These are different methods that might answer different research questions, as outlined by Morey et al. [27]. However, here, we used all methods for deriving stopping criteria and decisions about efficacy or futility.

Our simulations showed that in an experimental setting typical for current experimental biomedicine, if the effect exists, group sequential designs have lower costs because of early stopping for futility or efficacy (Table 1). With a large true effect size (d = 1) and *n* = 18 per group, sequential analyses that stop for significance reduce the costs up to 20%, while the power of these analyses do not differ from the traditional block design. Underpowered studies (d = 0.5 scenarios, Table 1) show only approximately 30% power for classical as well as sequential approaches, while the reduction in costs through sequential design is minor. This stresses the need for sufficiently powered studies even with sequential analyses. As expected, average effect sizes among successful experiments are overestimated in the traditional approach and slightly more so in the sequential design. Larger experiments that can stop for both success and futility show a similar pattern: sequential analysis has similar power as the traditional approach, while costs are reduced substantially.

## Efficiency versus predictive ability in a real-world setting

**Fig 2:**
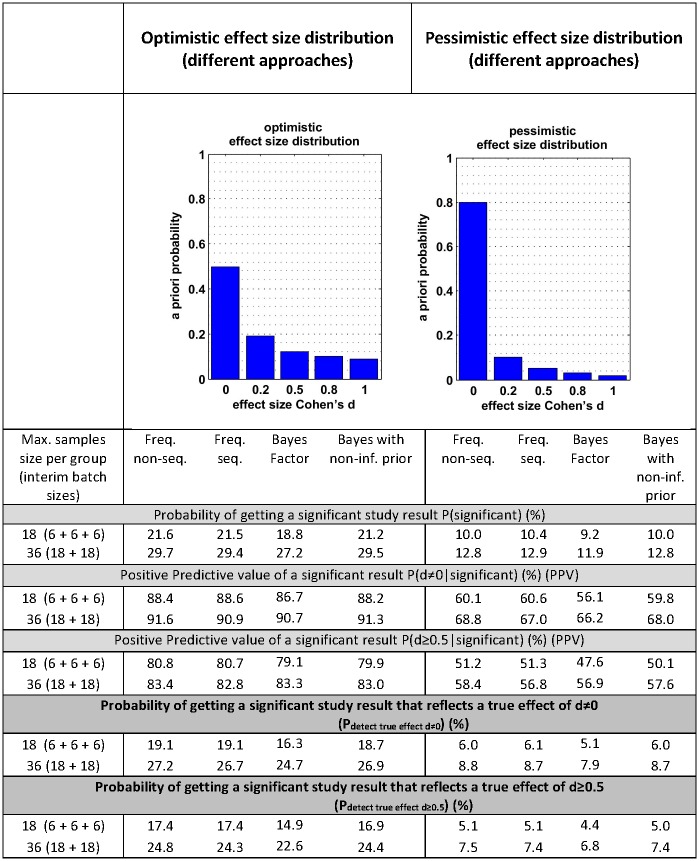
Predictive capabilities of sequential designs compared to traditional nonsequential design for two different scenarios of potential effect size distributions. Upper left: “optimistic” scenario with more large effect sizes. Upper right: “pessimistic” scenario with mostly effect sizes of 0. Bottom: Probability of getting a significant test result reflecting a true effect of d ≠ 0 or d ≥ 0.5, respectively, for the two different scenarios of effect size distributions. First, the probabilities P(significant) for getting any significant study results are given, then the corresponding positive predictive values, and, finally, the product of both giving the corresponding overall probability of getting a significant study result that truly represents an effect of d ≠ 0 or d ≥ 0.5 (P_detect true effect_). Stopping rules that allowed early stopping for futility or success as given in Table 1.

The simulations above differ from the real-world setting where we, despite setting out to detect an effect beyond a certain (biological) threshold, never know the true effect size a priori. In another set of simulations, we therefore assumed a specific distribution of true effect sizes within the universe of studies that can be performed. Such distributions may vary in different fields of research. This is relevant because, as with different effect size distributions and the chance of early stopping an experiment, the predictive probability of a “statistically significant” signal, i.e., the probability that a significant result really reflects a true effect, is different. To understand the ability to predict in a real-world setting, we simulated analyses with two different distributions of effect estimates: one optimistic and one pessimistic (Fig 2, S1 Fig). Through these simulations, we estimated the probabilities of obtaining an effect of any size d > 0 or at least size d ≥ 0.5 for both the traditional frequentist approach and group sequential designs. Overall, there are no major differences in these probabilities between the traditional and sequential approaches—despite the fact that the latter uses fewer animals. More importantly, this table shows that the main driver behind these probabilities is the a priori distribution of effect sizes (optimistic versus pessimistic).

## Applications of sequential designs

To the best of our knowledge, there are no groups or programs currently implementing sequential designs in preclinical experimental studies evaluating the efficacy of treatments or interventions. However, we are aware that the practice of interim analyses is applied informally when a statistically significant effect is desired but not found, and the analyses are rerun until significance has been achieved (a practice known as “p-hacking”[28]). Clearly, this practice inflates false-positive rates, as it violates the preset type I error (α–error) probability by not accounting for multiple testing in these unplanned interim analyses [10].

Despite the benefits suggested by our simulations, sequential approaches have properties that may limit their application in preclinical experimental biomedicine. The clearest disadvantage of group sequential designs is that each next stage can only be started after the outcome of the preceding stage is fully assessed and analyzed. Sequential analysis may require additional resources to set up, regulate, and monitor the independence of interim analyses, as well as additional statistical expertise. Another consideration is that a step-by-step design might increase the impact of batch and learning effects. However, the largest obstacle might be lack of familiarity with these methods in the field and amongst animal ethics committees, editorial boards, and peers. With this paper, we aim to spur the discussion and stimulate others to consider using sequential designs to increase the efficiency of their studies. Moreover, if in vivo researchers are to get ethical approval for this approach from their various committees, this article might help persuade those committees.

We posit that a substantial number of experiments in preclinical biomedicine can be planned and executed with batch sizes and sufficiently short intervals between treatments and outcome assessments to render them amenable to group sequential design–based methods (for an example, see S2 Text). Sequential designs can lead to a substantial reduction in animal resource. When these savings are invested in increased sample sizes (which, paradoxically, may not be higher than the current ones), sequential designs have the potential to increase the predictive ability of preclinical biomedical experiments and to reduce the current unacceptable levels of waste due to underpowered studies.

**Box 1. Points to consider when planning a group sequential design study**

Planning a study design as a group sequential design requires considerations before starting the study (see [17]; [18]):

**Type of adaptive design**

- Group sequential design is one simple type of adaptive design, in which the sample size is adapted during the study.
- Other types of adaptive design, such as designs with sample size reestimation, adaptive dose-response designs, treatment selection designs, or adaptive randomization designs, should be considered as well.

**Feasibility**

Is it feasible for the planned study:

- to plan larger sample sizes than for fixed designs with the same power (even if the expected sample size in case of an effect might be lower than for fixed designs)?
- to include additional time for the interim analysis? How many interim steps, and at which points, are feasible?

**Preplanning**

This includes:

- clearly specified hypotheses (adaptation should not be done with regard to generating hypotheses in confirmatory studies),
- decisions about reasons for early stopping: because of efficacy, futility, or both (stopping for futility is more important for larger studies),
- decisions about stopping criteria to reject the null hypothesis/or stop because of futility at each stage (related to power, type I error, frequentist or Bayesian kind of analysis, number of stages, sample size at each stage), and
- sample size estimation (depending on kind of statistical test, power, type I error, assumed effect size, number of stages, stopping criteria).

**Type I error (frequentist approach) [19]**

- Because of multiple testing, type I error is inflated, but different methods of alpha-adjustment ensure an overall type I error rate of 0.05:
- Pocock [16]: same significance level at each stage (e.g., three stages (two interim analyses): α = 0.0221 at each stage) (disadvantage: low level at the final stage, which makes it more difficult to get a significant result).
- O’Brien–Fleming [11]: significance level is very conservative at early stages and almost 0.05 at the final stage (e.g., three stages: α_1_ = 0.0006, α_2_ = 0.0151, α_3_ = 0.0471) (advantage: almost 0.05 at the final stage).
- Haybittle–Peto [20,21]: at all interim stages α_i_ = 0.001, at the final stage: α_final_ = 0.05 (advantage: easy to implement and understand and 0.05 level at the final stage, disadvantage: hard to stop early).
- Other more flexible approaches with regard to sample size at stages are also possible (using alpha-spending functions [22]).

**Bayesian approaches [23]**

- Points of consideration with regard to type of design, feasibility, and preplanning are similar to designs with frequentist approaches.
- Type I error normally is not of importance in Bayesian frameworks.
- But, regulatory authorities (e.g., [24]) expect evaluation of type I error also for Bayesian statistics.

**Software** for deriving and describing group sequential designs (including power considerations and sample size estimation):

- R package gsDesign (frequentist approach, [25])
- R package gsbDesign (Bayesian approach, [26])

## Supporting information

S1 Fig Predictive capabilities of sequential design (Pocock boundaries). (PDF)

S1 Table Early stopping for significance or futility using sequential group sequential design with Pocock-boundaries. (DOCX)

S1 Text Supporting materials and methods. (DOCX)

S2 Text Illustrative example comparing conventional and group sequential designs using real experimental data from a pre-clinical study in mice. (DOCX)
