# Supplementary material for: Increasing efficiency of preclinical research by group sequential designs
Source: PLoS Biol. 2017 Mar 10;15(3):e2001307. doi: 10.1371/journal.pbio.2001307 (PMC5345756; doi:10.1371/journal.pbio.2001307)
Supplement: S1 Fig — (PDF) [file pbio.2001307.s001.pdf]

S3 Figure: Predictive capabilities of sequential design (Pocock boundaries).

	**Optimistic effect size distribution (different approaches)**	**Pessimistic effect size distribution (different approaches)**
	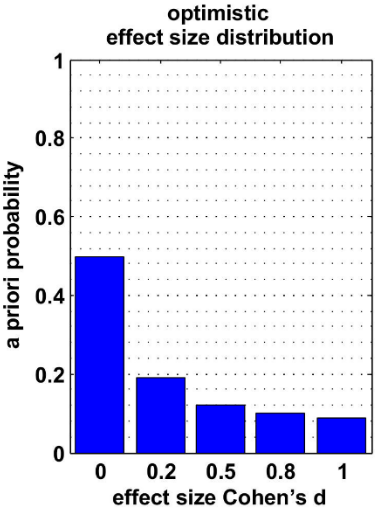	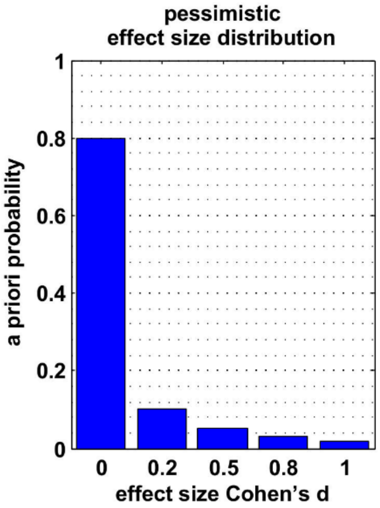
Max. samples size per group (interim batch sizes)	Freq. seq. Pocock	Freq. seq. Pocock
Probability of getting a significant study result P(significant) (%)		
18 (6 + 6 + 6)<br>36 (18 + 18)	19.3<br>28.0	9.4<br>12.2
Positive Predictive value of a significant result P(d≠0|significant) (%) (PPV)		
18 (6 + 6 + 6)<br>36 (18 + 18)	86.5<br>90.9	55.9<br>66.7
Positive Predictive value of a significant result P(d≥0.5|significant) (%) (PPV)		
18 (6 + 6 + 6)<br>36 (18 + 18)	78.7<br>83.3	47.2<br>57.1
**Probability of getting a significant study result that reflects a true effect of d≠0 ($P_{detect\ true\ effect\ d\neq0}$) (%)**		
18 (6 + 6 + 6)<br>36 (18 + 18)	16.7<br>25.4	5.3<br>8.2
**Probability of getting a significant study result that reflects a true effect of d≥0.5 ($P_{detect\ true\ effect\ d\geq0.5}$) (%)**		
18 (6 + 6 + 6)<br>36 (18 + 18)	15.2<br>23.3	4.5<br>7.0

Upper Left: 'Optimistic' scenario with more larger effect sizes. Upper right: 'Pessimistic' scenario with mostly effect sizes of 0. Bottom: Probability of getting a significant test result reflecting a true effect of d ≠0 or d≥0.5, respectively. First, the probabilities P(significant) for getting any significant study results are given, then the corresponding positive predictive values and finally the product of both giving the corresponding overall probability of getting a significant study result that truly represents an effect of d≠0 or d≥0.5 ($P_{detect\ true\ effect}$). Stopping rules that allowed early stopping for futility or success as given in Table S1.
