# Supplementary material for: Increasing efficiency of preclinical research by group sequential designs
Source: PLoS Biol. 2017 Mar 10;15(3):e2001307. doi: 10.1371/journal.pbio.2001307 (PMC5345756; doi:10.1371/journal.pbio.2001307)
Supplement: S1 Table — (DOCX) [file pbio.2001307.s002.docx]

***S1 Table: Early stopping for significance or futility using sequential group sequential design with Pocock-boundaries [1] (examples with n=36 or n=72)***

|  | small study (n=36) stop for significance, 3 stages | | Larger study (n=72) stop for significance or futility, 2 stages | | |
| --- | --- | --- | --- | --- | --- |
|  | Sample size (per group) | **Freq.**  **seq.**  **Pocock** | | Sample size (per group) | **Freq.**  **seq.**  **Pocock** |
| **d=0** | | | | | |
| **Stage 1** [%] sign. /futility | 12 (6 vs. 6 ) | 2.4 | | 36 (18 vs. 18) | 3.2/50.7 |
| **Stage 1+2** [%] sign. | 24 (12 vs. 12) | 3.9 | | - |  |
| **Stage 1+2(+3)=type 1 error** [%] sign. | 36 (18 vs. 18) | **5.2** | | 72 (36 vs. 36) | **5.1** |
| **Cost** [mean number of animals] |  | 36 | |  | 53 |
| **d_est_** |  | 1.31 | |  | 0.79 |
| **d=0.5** | | | | | |
| **Stage 1** [%] sign. /futility | 12 (6 vs. 6 ) | 6.8 | | 36 (18 vs. 18) | 23.6/18.8 |
| **Stage 1+2** [%] sign. | 24 (12 vs. 12) | 16.1 | | - |  |
| **Stage 1+2(+3)** **=Power** [%] sign. | 36 (18 vs. 18) | **25.7** | | 72 (36 vs. 36) | **48.5** |
| **Cost** [mean number of animals] |  | 33 | |  | 57 |
| **d_est_** |  | 1.12 | |  | 0.79 |
| **d=1.0** | | | | | |
| **Stage 1** [%] sign. /futility | 12 (6 vs. 6 ) | 22.0 | | 36 (18 vs. 18) | 76.7/0.9 |
| **Stage 1+2** [%] sign. | 24 (12 vs. 12) | 52.9 | | - |  |
| **Stage 1+2(+3)** **=Power** [%] sign. | 36 (18 vs. 18) | **75.1** | | 72 (36 vs. 36) | **97.5** |
| **Cost** [mean number of animals] |  | 27 | |  | 44 |
| **d_est_** |  | 1.28 | |  | 1.03 |

***Simulations based on a total number of 18 or 36 samples per group. Power or type I error for three different standardized effect sizes Cohen’s d =0 / 0.5 / 1.0, respectively.*** *Numbers give* ***cumulative*** *percentages of statistically significant trials in percent [%] out of 10,000 simulation runs, as well as ‘Costs’ defined as the long term mean of experimental units, and median estimated effect sizes in significant trials (d_est_).* ***Small study with n=18 per group:*** *Stage 1: n=12 (6 vs. 6), stage1+2: n=24 (12 vs. 12), stage 1+2+3: n=36 (18 vs. 18) experimental units. Stopping rules that allowed early stopping: Frequentist sequential (Pocock): significance levels for interim analyses: α_1_=α_2_=α_3_=0.0221 according to [16].*

***Larger study with n=36 per group:*** *Stage 1: n=36 (18 vs. 18), stage1+2: n=72 (36 vs. 36) experimental units. Stopping rules that allowed early stopping for futility or significance: Frequentist sequential (Pocock)[16]:* α_futility_= 0.5, α_1_=α_2_=0.0295*.*

*All sequential approaches used were calibrated to get a type I error of about 5%.*

**References**

1. Pocock SJ. Interim analyses for randomized clinical trials: the group sequential approach. Biometrics. 1982;38(1):153-62. Epub 1982/03/01. PubMed PMID: 7082757.
