# Supplementary material for: Increasing efficiency of preclinical research by group sequential designs
Source: PLoS Biol. 2017 Mar 10;15(3):e2001307. doi: 10.1371/journal.pbio.2001307 (PMC5345756; doi:10.1371/journal.pbio.2001307)
Supplement: S1 Text — (DOCX) [file pbio.2001307.s003.docx]

**S1_Text: Materials and Methods**

**Search strategy for text mining**

Using text mining of full-text articles in a subset of the PubMed Central Open Access database we investigated the prevalence of group sequential designs in experimental, pre-clinical biomedical research utilizing rats or mice.

Using PubMed we searched in June 2016 for articles published between 1st January, 2010 and 31st December, 2014 using the following query: "Drug Evaluation, Preclinical"[Mesh] AND ("Mice"[Mesh] OR "Rats"[Mesh]) AND treatment AND (Journal Article[ptyp] NOT Review[ptyp]) AND ("2010/01/01"[PDAT] : "2014/12/31"[PDAT]) AND English[lang] AND pubmed pmc[sb]. The returned PubMed Central (PMC) reference numbers for articles were used to obtain full-text documents from the PMC Open Access subset as of February 2015, which contains over one million full-text articles to date.

A rule-based text mining approach was developed to identify the keywords ‘interim analysis’, ‘Bayes’ (to include Bayes or Bayesian), ‘sequential (analysis or design)’, ‘adaptive (allocation or design)’, and ‘termination rule’ in the full-text. GATE open source text mining software [1], was used for the mining process with the designed rule following its notation (Windows version 8.1).

**Overall design of the simulation study**

We used simulations to show how group sequential trial designs may improve the efficiency of typical pre-clinical experimental designs. We compared a standard non-sequential design analysed using a classical frequentist approach to group sequential designs analysed by both frequentist and Bayesian analysis methods with regard to their performance (i.e. power, sample size and characteristics of significant experiments).

We simulated two-group-trial data with sample sizes of 18 vs. 18 and 36 vs. 36 experimental units (Table 1), a continuous normally distributed outcome and three different standardized effect sizes between the two groups (Cohen’s d= 0, 0.5 and 1). For every scenario with regard to sample size and effect size we used 10,000 simulations to obtain estimations for probabilities for early stopping for the different stopping criteria described below. The scenarios with effect size d=0 (i.e. the Null-hypothesis (H0) is true - there is no difference between the groups) were used to calibrate every approach not to exceed the typical type I error rate of 0.05. To evaluate the power of the different analysis designs we considered a medium effect size of d = 0.5 (i.e. the difference in means equals half the common standard deviation), as well as a large effect size of d=1 (i.e. the difference in means equals the common standard deviation). The sequential designs were not a priori designed to have the same power for d>0 scenarios as the non-sequential designs. A priori sample size calculation revealed that 18 samples per group are required to detect an effect size of d=1 at a two-sided type I error level of α = 0.05 and a power of 83%. Such group sizes is substantially greater than is common seen in pre-clinical research. With a group size of 36 experimental units (total n=72) effect sizes of 0.67 could be shown (with power=80% and two-sided α = 0.05).

In a first set of simulations, we used a study design that allowed 2 interim analyses after recruitment of 6 animals per group (1. “interim analysis at stage 1”) and after a sum of 12 animals per group (2. “interim analysis at stage 2”). After each stage we checked whether the stopping criteria specified below for termination of the study were met (Table 1). If so, the simulation run stopped, if not, the next stage was added. In a second set of simulations we used 18 animals per group for the first interim analysis and a sum of 36 samples per group for the second (final) analysis (Table 1). Though incredibly large for actual pre-clinical studies, this larger sample size (total n=72) allowed us to consider additional stopping rules for futility. For each set of simulations, we also compared the group sequential designs to a non-sequential analysis protocol. Efficiency was measured by the number of experiments stopped early based on significant difference between groups. Simulation results were compared with regard to type I error rate (false positives) and statistical power.

**Frequentist group sequential design**

In clinical research a type I error of 0.05 is well established. If multiple statistical tests are conducted in the same study, the risk of type I error increases [2]. To overcome the problem of multiple testing, several procedures to adjust the significance level have been recommended to achieve an overall type I error of 0.05 [3-6]. Here we used the commonly applied O’Brien Fleming alpha levels for interim analyses [7] that leaves the significance level of the final analysis approximately at the traditional 0.05 at the cost of very strict alpha levels for the interim analyses. The t-test for two independent samples was used to compare group means with the specified stopping criteria after stage 1 α_1_=0.0006, after stage 2 α_2_=0.0151 and after stage 3 α_3_=0.0471. A p-value lower than α_i_ was considered significant and the simulation run stopped. For the second set of simulations with 36 animals per group and only one interim analysis, stopping criteria were α_1_=0.0065, α_2_=0.0525. Additionally, stopping rules for futility were considered to avoid spending resources if the probability to accept the null hypothesis of no effect was high, α_futility_= 0.5 (stop if p>0.5), in interim analysis. Additionally, the same scenarios using Pocock-boundaries (equally sized alpha level at each stage) can be found in the supplementary Table S1 (S1_Table.doc; Early stopping for significance or futility using sequential group sequential design with Pocock-boundaries) .

**Bayesian methods**

In Bayesian frameworks the analysis depends not only on the data at hand but also on prior assumptions and beliefs about the analysed effect. These prior assumptions are modelled with a prior probability distribution that reflects mathematically the extent, and the grade of uncertainty about this extent, of the analysed effect and affects the results of the analysis [8]. To avoid the criticism of using prior beliefs that could potentially be wrong, one common strategy in Bayesian statistics is to use so-called non-informative or sceptical priors [8]. Here, a flat probability distribution (uniform distribution) was used as an non-informative prior for the effect size, assuming that every possible effect size has the same probability and a Jeffrey’s prior for the variance where the probability of variance σ^2^ is inverse to σ^2^  [9] (see supporting information online https://figshare.com/articles/additional_information_to_Increasing_Efficacy_of_Pre-clinical_Research_by_Group_Sequential_Designs/4569577). Limits of the credible intervals were selected manually such that type I error rate of 0.05 was not exceeded.

The software OpenBUGS (version 3.2.3) and the R packages R2WinBUGS 2.1-19 were used to calculate credible intervals for the difference of means between the two groups. The R code used is given online (https://figshare.com/articles/additional_information_to_Increasing_Efficacy_of_Pre-clinical_Research_by_Group_Sequential_Designs/4569577).

**Scenarios of potential real-world effect size distributions- predictive values of a significant test result**

Normally, the true effect size of an intervention is not known a priori, thus the benefit of a group sequential design is also a priori not known. In the whole “universe” of trials different effect sizes are present with different frequencies. In order to estimate the predictive value of a significant study result within the sequential analysis, we assumed two different scenarios of realistic effect size distributions. One scenario is optimistic assuming the probability of an effect size of at least d=0.5 to be 31% (d=0: 50.0%; d=0.2: 18.3%; d=0.5: 13.5%; d=0.8: 10.0%; d=1: 8.2%; left column, Figure 2). The other is more pessimistic assuming the probability of obtaining data with an effect size of at least 0.5 to be only 10% (d=0: 80.0%; d=0.2: 10.0%; d=0.5: 5.0%; d=0.8: 3.0%; d=1: 2.0%, right column, Figure 2). For this analysis we performed the simulations for d=0, 0.2, 0.5, 0.8 and 1.0. Figure 2 gives the percentage of simulated trials that became significant according to the stopping criteria defined above; which is equivalent to the probability of getting a significant test results at any stage of the sequential analysis. The corresponding positive predicted value (PPV, probability that a significant result reflects a true effect of d>0 or d≥0.5) was then calculated as the sum of the products of the specific power multiplied by the probability of a specific effect size over all possible effect sizes (either d>0 or d≥0.5). Finally, the product of the PPV and the probability of obtaining a significant test result at any stage of the sequential analysis gives the corresponding overall probability of obtaining a significant study result that represents an effect of d>0 or d≥0.5 (P_detect true effect_ _d≥0.5_), respectively.

**References Supplemental Material**

1. Cunningham H, Tablan V, Roberts A, Bontcheva K. Getting more out of biomedical documents with GATE's full lifecycle open source text analytics. PLoS Comput Biol. 2013;9(2):e1002854.

2. Sainani KL. The problem of multiple testing. PM&R. 2009;1(12):1098-103.

3. Bender R, Lange S. Adjusting for multiple testing—when and how? Journal of clinical epidemiology. 2001;54(4):343-9.

4. Dmitrienko A, Bretz F, Westfall PH, Troendle J, Wiens BL, Tamhane AC, et al. Multiple testing methodology. Multiple testing problems in pharmaceutical statistics. 2009:35-98.

5. Dmitrienko A, Tamhane AC, Bretz F. Multiple testing problems in pharmaceutical statistics: CRC Press; 2009.

6. Hommel G, Bretz F, Maurer W. Powerful short‐cuts for multiple testing procedures with special reference to gatekeeping strategies. Statistics in Medicine. 2007;26(22):4063-73.

7. O'Brien PC, Fleming TR. A multiple testing procedure for clinical trials. Biometrics. 1979:549-56.

8. Spiegelhalter DJ, Abrams KR, Myles JP. Bayesian approaches to clinical trials and health-care evaluation: John Wiley & Sons; 2004.

9. Lindley DV, editor The use of prior probability distributions in statistical inference and decision. Proc 4th Berkeley Symp on Math Stat and Prob; 1961.
