# Supplementary material for: Increasing efficiency of preclinical research by group sequential designs
Source: PLoS Biol. 2017 Mar 10;15(3):e2001307. doi: 10.1371/journal.pbio.2001307 (PMC5345756; doi:10.1371/journal.pbio.2001307)
Supplement: S2 Text — (DOCX) [file pbio.2001307.s004.docx]

**S2 Text. Illustrative example comparing conventional and group sequential designs using real experimental data from a pre-clinical study in mice**

To demonstrate how group sequential trial designs would affect the course and interpretation of results in a typical pre-clinical study we use real data from an experimental study of stroke in mice ([1], (original data given below, Table A). These data were analysed with group sequential approaches by assigning the mice to virtual batches and analysing the batches *post-hoc* as if a group sequential design was used. The original study investigated whether a putative neuroprotective compound reduces brain infarct sizes in a widely used experimental murine stroke model [1]. Mice received either saline or the compound dissolved in saline intra-peritoneally, starting 6 hours after transient occlusion of the middle cerebral artery for one hour. Three days afterwards, brain infarcts were quantified histologically. In the control group 2 mice had to be excluded due to mortality during surgery (i.e. before onset of treatment), resulting in a final sample size of 34. The study was conducted with a conventional frequentist design of batches of 18 and 18 mice, and was powered to detect an effect size of d=1 at α=0.05 and ß=0.2.

***Table A: Raw data (infarct volume in mm^3^) from illustrative example [1]: 18 versus 16 mice (2 drop outs)***

|  | **control** | **intervention** |
| --- | --- | --- |
| **Stage1** | 91.5 | 40.7 |
|  | 71.2 | 53.8 |
|  | 64.9 | 44.9 |
|  | 54.8 | 72.6 |
|  | 62.7 | 50.6 |
|  | -- | 49.2 |
| Sample size (stage 1) | 5 | 6 |
| Mean (SD) | 69.0 (13.8) | 52.0 (11.1) |
| **Stage2** | 26.9 | 27.3 |
|  | 111.1 | 22.8 |
|  | 72.9 | 50.7 |
|  | 107.4 | 77.9 |
|  | 81.4 | 58.4 |
|  | 93.9 | 70.5 |
| Sample size (stage 1+2) | 11 | 12 |
| Total Mean (SD) | 76.2 (24.5) | 51.6 (16.9) |
| **Stage 3** | 54.0 | 44.5 |
|  | 59.8 | 75.4 |
|  | 63.5 | 35.3 |
|  | 105.3 | 61.5 |
|  | 58.0 | 49.4 |
|  | -- | 85.9 |
| Sample size (stage 1+2+3) | 16 | 18 |
| total Mean (SD) | 73.7 (23.1) | 54.0 (17.5) |

We compare the results originally derived using a conventional frequentist block design to an analysis that could have been done if the same data came from a group sequential design (Table B). In this pre-clinical study, mice were treated with a putative neuroprotectant 6 hours after transient occlusion of the middle cerebral artery for 1 hour. Had a group sequential design been used (using either frequentist or Bayesian analysis methods), the study could have stopped early due to successfully demonstrating that in the intervention group infarct sizes were significantly smaller than in the control group, after using 23 instead of 34 mice. The estimated difference in stroke volume would have been modestly larger in the sequential scenario (24.6 mm^3^) than in the frequentist, non-sequential trial (20.3 mm^3^).

***Table B: Illustrative example of a pre-clinical study in mice****. The intervention aimed at reducing brain infarct volume after experimental middle cerebral artery occlusion. Comparison of frequentist non-sequential, frequentist sequential, and Bayesian analysis with non-informative prior (powered for effect size d= 1.0 with alpha = 0.05, power = 0.8). Significance levels for interim analyses: α_1_=0.0006, α_2_=0.0151 according to [7]. Credible intervals (CRI) for difference in means: CRI stage 1: 99.8%, stage 2+3: 96.8%. If 0 is not included in specific CRI, then study was stopped for success*. *Significant results are marked in bold. Note that 2 mice had to be excluded from the control (crtl) group due to death during surgery.*

|  |  |  | **approach and test result** | | |
| --- | --- | --- | --- | --- | --- |
|  | Sample size  (ctrl vs. intervention) | Mean (SD) stroke volume  for control / intervention [mm^3^] | **Freq.**  **non-seq.**  p-value | **Freq.**  **seq.**  p-value | **Bayes with**  **non-inf. Prior**  CRI |
|  | | | | | |
| **Stage 1** | 11 (5 vs. 6 ) | 69.0 (13.8) /  52.0 (11.1) | - | 0.049 | (-47.9, 11.2) |
| **Stage 1+2** | 23 (11 vs. 12) | 76.2 (24.5) /  51.6 (16.9) | - | **0.010** | **(-44.0,-4.6)** |
| **Stage 1+2+3** | 34 (16 vs. 18) | 73.7 (23.1) /  54.0 (17.5) | **0.008** |  |  |

**References**

1. Engel O, Kolodziej S, Dirnagl U, Prinz V. Modeling stroke in mice-middle cerebral artery occlusion with the filament model. J Vis Exp. 2011;47:2423.
